# 1-(4-Methyl­benzene­sulfon­yl)-1-phenyl-1*H*,2*H*-cyclo­buta[*c*]quinoline

**DOI:** 10.1107/S2414314625010193

**Published:** 2025-11-28

**Authors:** Dieter Schollmeyer, Benjamin Dassonneville, Heiner Detert

**Affiliations:** a University Mainz, Duesbergweg 10-14, 55099 Mainz, Germany; b University of Mainz, Department of Chemistry, Duesbergweg 10-14, 55099 Mainz, Germany; Goethe-Universität Frankfurt, Germany

**Keywords:** crystal structure, hydrogen bridge, heterocycle, cyclo­butene, 2 + 2 cyclo­addition, 1,5-sulfonyl shift

## Abstract

The title compound, C_24_H_19_NO_2_S, forms strands along the *b*-axis direction *via* hydrogen bonds from the tolyl group to the sulfonyl oxygen atoms. The tricyclic framework is almost planar and the two six-membered aromatic substituents are nearly coparallel.

## Abstract 2

The title compound forms strands along the *b*-axis direction *via* hydrogen bonds from the tolyl group to the sulfonyl oxygen atoms. The tricyclic framework is almost planar and the two six-membered aromatic substituents are nearly coparallel.

## Graphical abstract

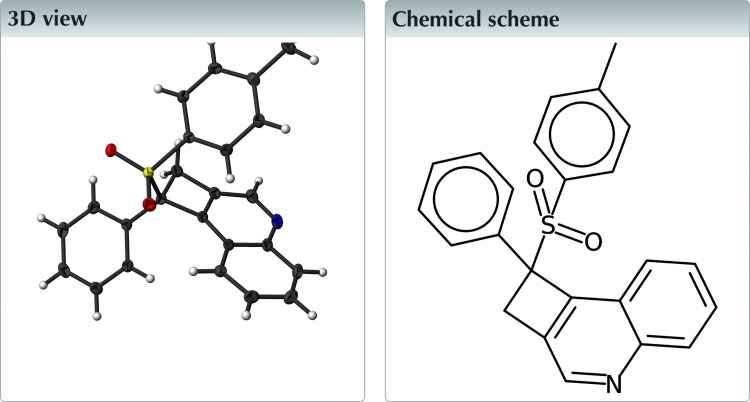

## Structure description

**Figure 1:**
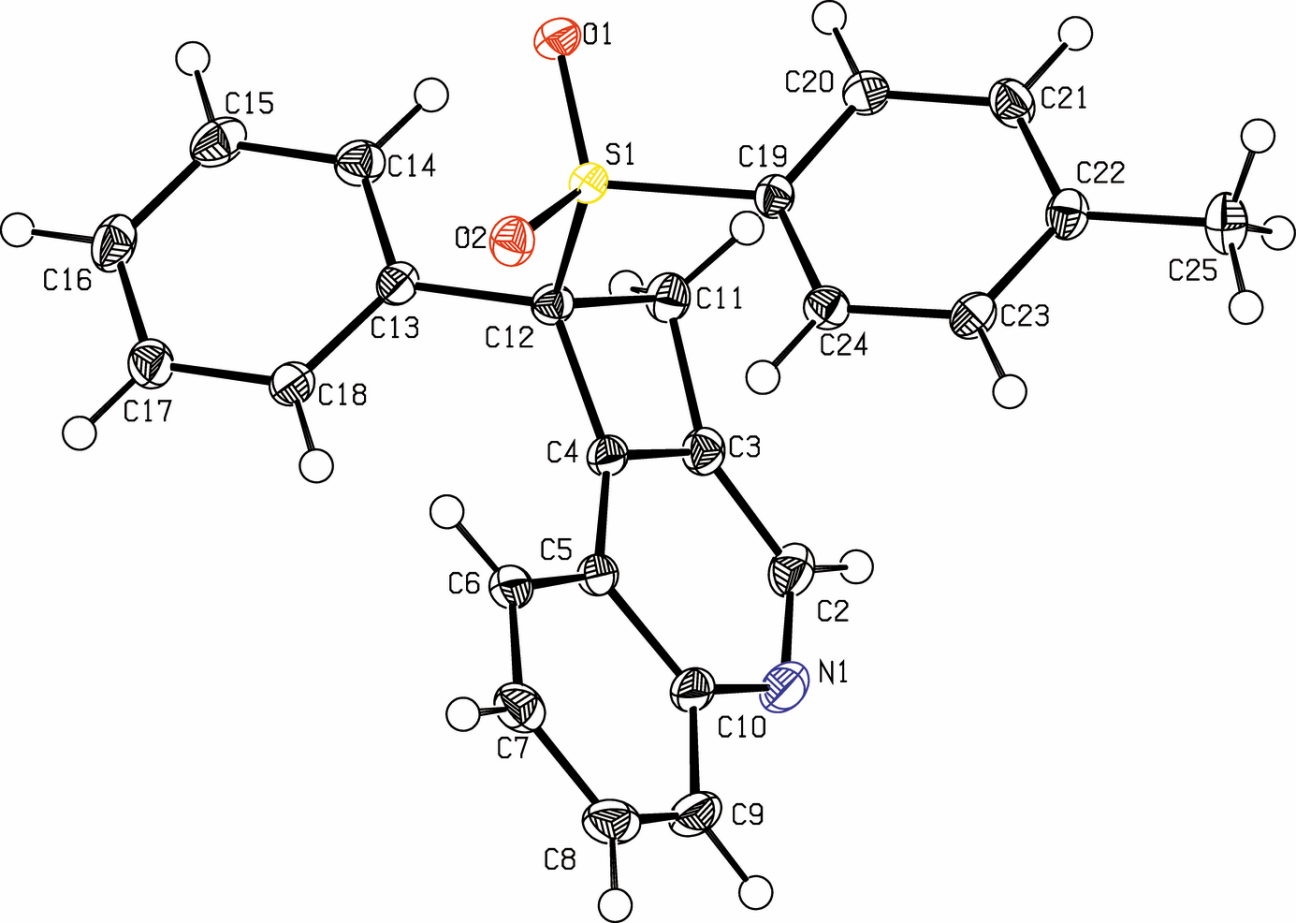
View the title compound. Displacement ellipsoids are drawn at the 50% probability level.

**Figure 2:**
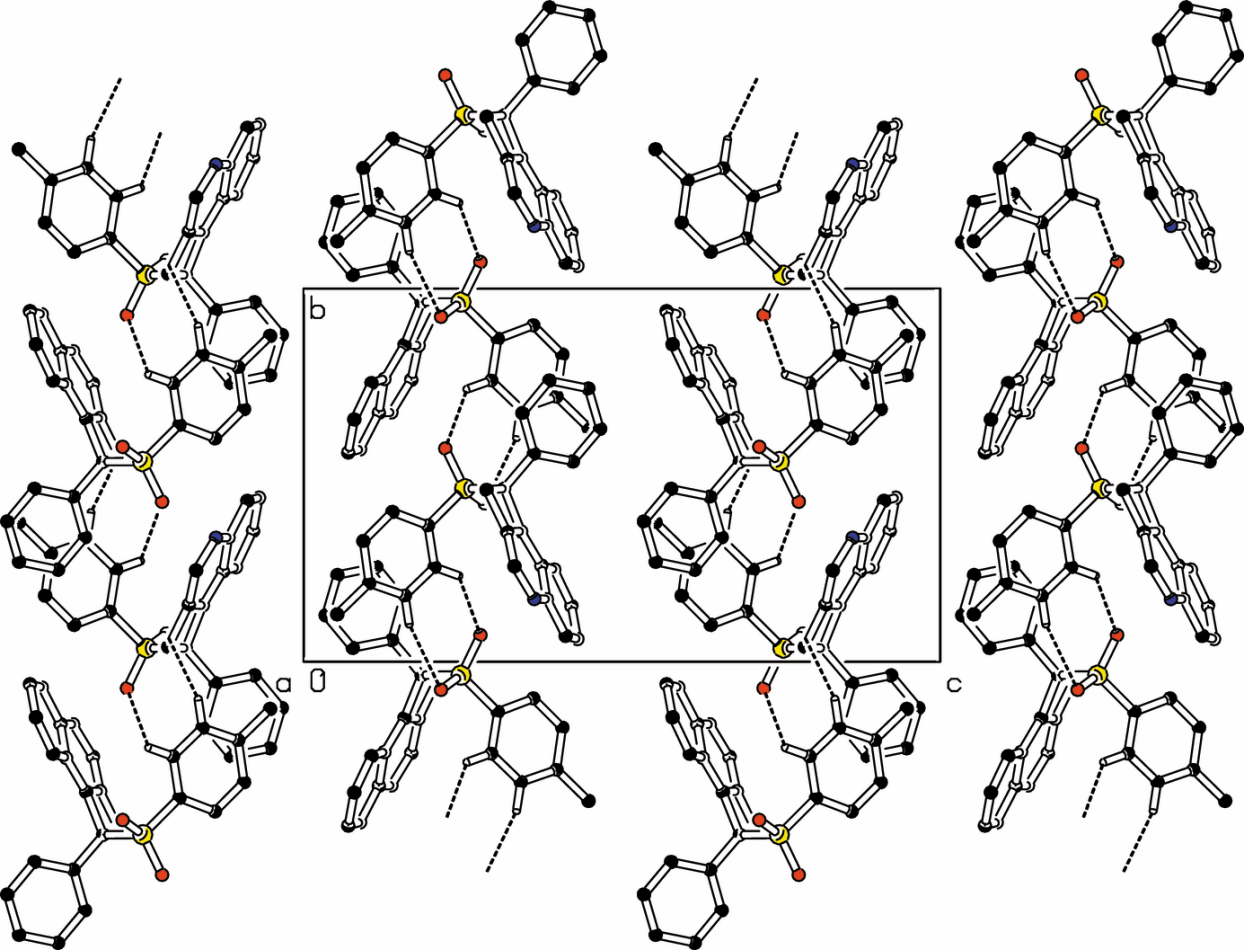
Part of the packing diagram. View along the *a*-axis direction. Hydrogen bonds are shown as dashed lines.

The title compound, C_24_H_19_NO_2_S (Fig. 1 ▸), was prepared in a larger project on indolo-annulated heterocycles (Dassonneville *et al.*, 2023 ▸; Limbach *et al.*, 2018 ▸; Letessier *et al.*, 2013 ▸). It is an isomer and follow-up product of the recently reported *N*-propargyl-*N*-tosyl­amino­tolane (Dassonneville *et al.*, 2025 ▸). The cyclo­butene ring and the quinoline system include a dihedral angle of 5.04 (9)°, C11 [0.159 (2) Å] and C12 [0.026 (2) Å] lie below the quinoline plane. The inter­planar angle between the quinoline system and the phenyl ring is 57.42 (6)°, the latter is almost parallel to the tolyl unit. The inter­planar angle is only 6.03 (7)°. The bond angles in the cyclo­butene unit are larger at the *sp*^3^-carbon atoms (C11—C3—C4: 94.75 (13)°, C3—C4—C12: 93.72 (13)° than on the *sp*^2^-carbon atoms: C4—C12—C11: 86.19 (11)° and C12—C11—C3: 85.29 (11)°. The crystal packing features chains along the *b*-axis direction. The mol­ecules are connected *via* hydrogen bonds (Table 1 ▸, Fig. 2 ▸) from the tolyl ring to the sulfonyl O atom, lengths are H23⋯O2: 2.52 Å and H24⋯O1: 2.47 Å, angles are C23—H23⋯O2: 161° and C24—H24⋯O1: 137°. A twofold screw axis ralates the mol­ecules geometrically.

## Synthesis and crystallization

**Figure 3:**
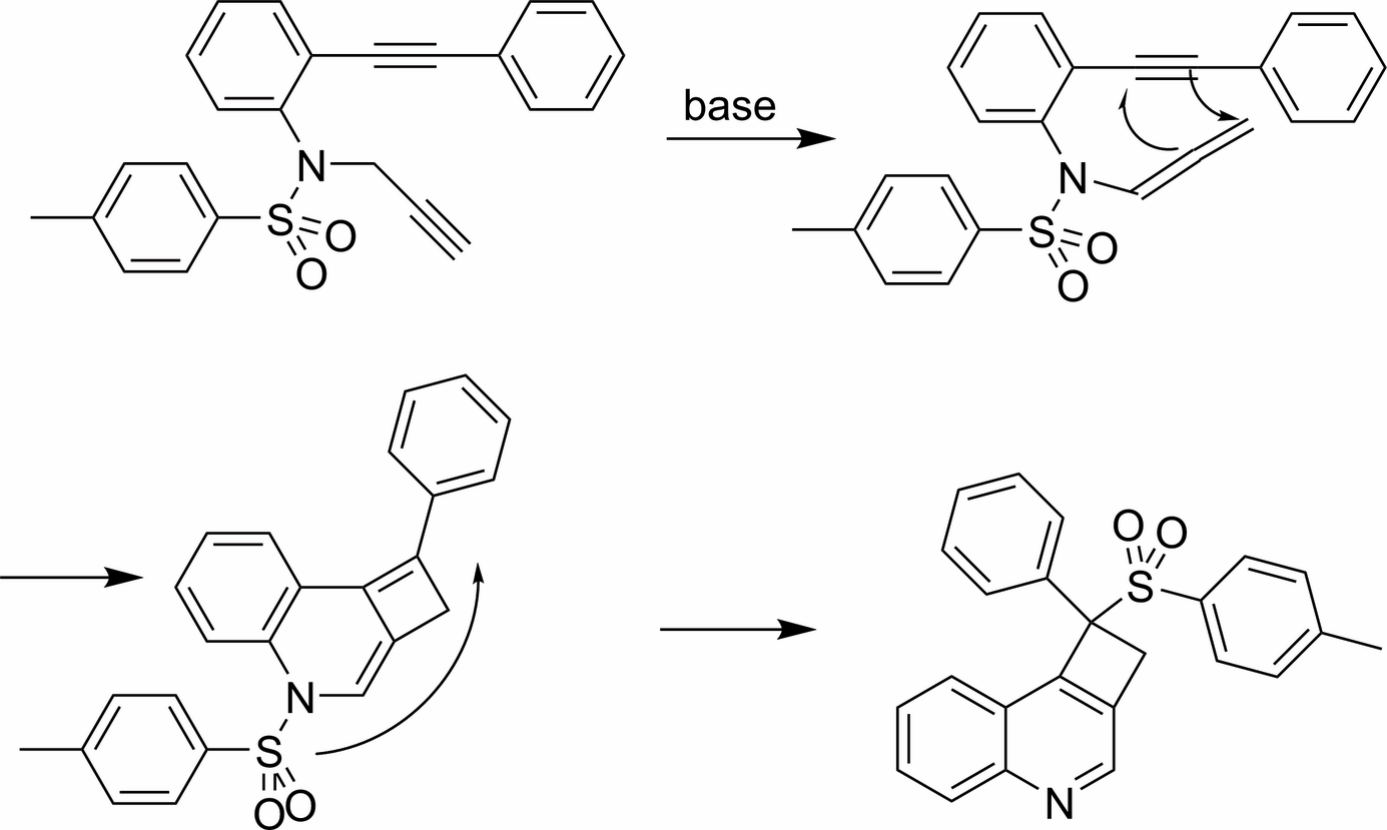
Possible synthetic mechanism.

The title compound, C_24_H_19_NO_2_S, appeared as by-product in low yield in the propargylation of 2-tosyl­amino­tolane (Dassonneville *et al.*, 2025 ▸). A possible mechanistic pathway is given in Fig. 3 ▸. A base-catalyzed isomerization of the propargyl unit to an allenyl substituent followed by 2 + 2 cyclo­addition of the outer double bond of the allene with the tolane triple bond generates a cyclo­butene with concomitant closure of the quinoline ring. A supra-supra­facial 1,5-shift of the sulfonyl group from quinoline-N to a cyclo­butene ring with aromatization of the pyridine ring gives the final compound. The compound crystallized from toluene solution as brownish crystals with m.p. = 426 K. The annotation of NMR signals follows IUPAC nomenclature. ^1^H-NMR (400 MHz, CDCl_3_): 8.66 (*s*, 1 H, 2-H quin); 8.20 (*dd*, *J* = 8 Hz, J′= 4 Hz), 8.16 (*dd*, *J* = 8 Hz, J′= 4 Hz) (5-H, 8-H quin), 7.80 (*ddd*, 1 H), 7.74 (*ddd*, 1 H) (6-H, 7-H, quin), 7.67–7.63 (*m*, 2 H, ph), 7.42–7.35 (*m*, 5 H, ph + tol), 7.09 (*d*, 2 H, 3-H, 5-H, tol), 4.20 (*d*, *J* = 16 Hz, CH_2_), 3.77 (*d*, *J* = 16 Hz, CH_2_), 2.37 (*s*, 3 H, CH_3_). ^13^C-NMR (100 MHz, CDCl_3_): 147.09 (C_q_), 147.32 (C_q_), 145.27 (C_q_), 144.75 (CH, C-2 quin), 136.44 (C_q_), 132.96 (C_q_), 132.32 (C_q_), 131.28 (CH, C-6 or C-7 quin), 130.11 (2 CH), 130.08 (2 CH), 129.22 (CH), 129.19 (2 CH), 129.02 (CH), 128.52 (CH), 128.32 (2 CH), 124.33 (CH, C-7 or C-6 quin), 123.97 (C_q_), 78.96 (C_q_), 42.26 (CH_2_), 21.66 (CH_3_).

## Refinement

Crystal data, data collection and structure refinement details are summarized in Table 2 ▸.

## Supplementary Material

Crystal structure: contains datablock(s) I, global. DOI: 10.1107/S2414314625010193/bt4189sup1.cif

Structure factors: contains datablock(s) I. DOI: 10.1107/S2414314625010193/bt4189Isup2.hkl

Supporting information file. DOI: 10.1107/S2414314625010193/bt4189Isup3.cml

CCDC reference: 2502744

Additional supporting information:  crystallographic information; 3D view; checkCIF report

Additional supporting information:  crystallographic information; 3D view; checkCIF report

## Figures and Tables

**Table 1:** Hydrogen-bond geometry (Å, °)

*D*—H⋯*A*	*D*—H	H⋯*A*	*D*⋯*A*	*D*—H⋯*A*
C23—H23⋯O2^i^	0.95	2.52	3.431 (2)	161
C24—H24⋯O1^i^	0.95	2.47	3.2357 (19)	137

Symmetry code: (i) 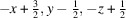.

**Table 2:** Experimental details

Crystal data	
Chemical formula	C_24_H_19_NO_2_S
*M*_r_	385.46
Crystal system, space group	Monoclinic, *P*2_1_/*n*
Temperature (K)	120
*a*, *b*, *c* (Å)	7.5268 (3), 12.0672 (7), 20.6402 (9)
β (°)	92.740 (3)
*V* (Å^3^)	1872.55 (16)
*Z*	4
Radiation type	Mo *K*α
μ (mm^−1^)	0.19
Crystal size (mm)	0.44 × 0.36 × 0.18

Data collection	
Diffractometer	STOE *IPDS* 2T
Absorption correction	Integration (*X-RED3*; Stoe & Cie, 2020 ▸)
*T*_min_, *T*_max_	0.933, 0.979
No. of measured, independent and observed [*I* > 2σ(*I*)] reflections	9143, 4455, 3856
*R*_int_	0.022
(sin θ/λ)_max_ (Å^−1^)	0.660

Refinement	
*R*[*F*^2^ > 2σ(*F*^2^)], *wR*(*F*^2^), *S*	0.042, 0.109, 1.08
No. of reflections	4455
No. of parameters	254
H-atom treatment	H-atom parameters constrained
Δρ_max_, Δρ_min_ (e Å^−3^)	0.49, −0.38

Computer programs 2.5.18.0 (Stoe & Cie, 2020 ▸), *SHELXT2014* (Sheldrick, 2015*a* ▸), *SHELXL2019/2* (Sheldrick, 2015*b* ▸) and *PLATON* (Spek, 2009 ▸).

## full crystallographic data

### 1-(4-Methylbenzenesulfonyl)-1-phenyl-1H,2H-cyclobuta[c]quinoline Crystal data

**Table:**

C_24_H_19_NO_2_S	*F*(000) = 808
*M**_r_* = 385.46	*D*_x_ = 1.367 Mg m^−^^3^
Monoclinic, *P*2_1_/*n*	Mo *K*α radiation, λ = 0.71073 Å
*a* = 7.5268 (3) Å	Cell parameters from 12708 reflections
*b* = 12.0672 (7) Å	θ = 2.6–28.4°
*c* = 20.6402 (9) Å	µ = 0.19 mm^−^^1^
β = 92.740 (3)°	*T* = 120 K
*V* = 1872.55 (16) Å^3^	Block, colorless
*Z* = 4	0.44 × 0.36 × 0.18 mm

### 1-(4-Methylbenzenesulfonyl)-1-phenyl-1H,2H-cyclobuta[c]quinoline Data collection

**Table:**

STOE IPDS 2T diffractometer	3856 reflections with *I* > 2σ(*I*)
Detector resolution: 6.67 pixels mm^-1^	*R*_int_ = 0.022
rotation method, ω scans	θ_max_ = 28.0°, θ_min_ = 2.6°
Absorption correction: integration (X-Red3; Stoe & Cie, 2020)	*h* = −9→9
*T*_min_ = 0.933, *T*_max_ = 0.979	*k* = −13→15
9143 measured reflections	*l* = −23→27
4455 independent reflections	

### 1-(4-Methylbenzenesulfonyl)-1-phenyl-1H,2H-cyclobuta[c]quinoline Refinement

**Table:**

Refinement on *F*^2^	Primary atom site location: dual
Least-squares matrix: full	Hydrogen site location: inferred from neighbouring sites
*R*[*F*^2^ > 2σ(*F*^2^)] = 0.042	H-atom parameters constrained
*wR*(*F*^2^) = 0.109	*w* = 1/[σ^2^(*F*_o_^2^) + (0.0478*P*)^2^ + 1.4388*P*] where *P* = (*F*_o_^2^ + 2*F*_c_^2^)/3
*S* = 1.08	(Δ/σ)_max_ = 0.001
4455 reflections	Δρ_max_ = 0.49 e Å^−^^3^
254 parameters	Δρ_min_ = −0.38 e Å^−^^3^
0 restraints	

### 1-(4-Methylbenzenesulfonyl)-1-phenyl-1H,2H-cyclobuta[c]quinoline Special details

**Table:**

Geometry. All esds (except the esd in the dihedral angle between two l.s. planes) are estimated using the full covariance matrix. The cell esds are taken into account individually in the estimation of esds in distances, angles and torsion angles; correlations between esds in cell parameters are only used when they are defined by crystal symmetry. An approximate (isotropic) treatment of cell esds is used for estimating esds involving l.s. planes.
Refinement. Hydrogen atoms were placed at calculated positions and were refined in the riding-model approximation with C_aromatic_–H = 0.95 Å, C_methyl_–H = 0.98 Å, C_methylene_–H = 0.99 Å, and with *U*_iso_(H) = 1.2 *U*_eq_(C) for aromatic and methylene H atoms and with *U*_iso_(H) = 1.5 *U*_eq_(C_methyl_). The methyl group was allowed to rotate but not to tip.

### 1-(4-Methylbenzenesulfonyl)-1-phenyl-1H,2H-cyclobuta[c]quinoline Fractional atomic coordinates and isotropic or equivalent isotropic displacement parameters (Å^2^)

**Table:**

	*x*	*y*	*z*	*U*_iso_*/*U*_eq_	
S1	0.64207 (5)	0.46384 (3)	0.25290 (2)	0.01500 (11)	
O1	0.63964 (16)	0.57122 (9)	0.22241 (5)	0.0202 (2)	
N1	0.2289 (2)	0.16806 (12)	0.36221 (7)	0.0245 (3)	
O2	0.80565 (15)	0.42439 (10)	0.28432 (5)	0.0199 (2)	
C2	0.1616 (2)	0.25542 (14)	0.33105 (8)	0.0228 (3)	
H2	0.039969	0.255983	0.316417	0.027*	
C3	0.2704 (2)	0.34742 (14)	0.31963 (7)	0.0187 (3)	
C4	0.4442 (2)	0.34911 (13)	0.34160 (7)	0.0165 (3)	
C5	0.5253 (2)	0.25768 (13)	0.37323 (7)	0.0172 (3)	
C6	0.7067 (2)	0.24665 (13)	0.39355 (7)	0.0190 (3)	
H6	0.786202	0.306822	0.388467	0.023*	
C7	0.7677 (2)	0.14939 (14)	0.42053 (8)	0.0231 (3)	
H7	0.890248	0.141725	0.433003	0.028*	
C8	0.6503 (3)	0.06065 (15)	0.42992 (8)	0.0267 (4)	
H8	0.694212	−0.005968	0.449289	0.032*	
C9	0.4743 (3)	0.06914 (14)	0.41153 (8)	0.0258 (4)	
H9	0.396757	0.008686	0.418641	0.031*	
C10	0.4059 (2)	0.16729 (13)	0.38190 (8)	0.0203 (3)	
C11	0.2768 (2)	0.46028 (13)	0.28729 (8)	0.0190 (3)	
H11A	0.198639	0.516693	0.305973	0.023*	
H11B	0.262576	0.458325	0.239374	0.023*	
C12	0.4775 (2)	0.46404 (12)	0.31482 (7)	0.0156 (3)	
C13	0.5228 (2)	0.55765 (13)	0.36154 (7)	0.0164 (3)	
C14	0.4825 (2)	0.66637 (14)	0.34362 (8)	0.0206 (3)	
H14	0.427011	0.681116	0.302206	0.025*	
C15	0.5227 (2)	0.75340 (14)	0.38585 (9)	0.0242 (3)	
H15	0.497528	0.827501	0.372878	0.029*	
C16	0.5998 (2)	0.73204 (15)	0.44701 (8)	0.0249 (4)	
H16	0.625610	0.791420	0.476187	0.030*	
C17	0.6390 (2)	0.62422 (15)	0.46546 (8)	0.0216 (3)	
H17	0.691211	0.609713	0.507426	0.026*	
C18	0.6024 (2)	0.53691 (13)	0.42282 (8)	0.0183 (3)	
H18	0.631596	0.463158	0.435420	0.022*	
C19	0.5675 (2)	0.36514 (13)	0.19515 (7)	0.0156 (3)	
C20	0.4723 (2)	0.40126 (13)	0.13940 (8)	0.0191 (3)	
H20	0.446551	0.477642	0.132914	0.023*	
C21	0.4162 (2)	0.32307 (14)	0.09379 (8)	0.0208 (3)	
H21	0.351596	0.346458	0.055557	0.025*	
C22	0.4529 (2)	0.21033 (14)	0.10284 (8)	0.0188 (3)	
C23	0.5476 (2)	0.17718 (13)	0.15898 (8)	0.0182 (3)	
H23	0.572953	0.100793	0.165659	0.022*	
C24	0.6058 (2)	0.25370 (13)	0.20536 (8)	0.0174 (3)	
H24	0.670775	0.230324	0.243521	0.021*	
C25	0.3904 (2)	0.12684 (15)	0.05270 (8)	0.0255 (4)	
H25A	0.443051	0.143849	0.011332	0.038*	
H25B	0.260469	0.129811	0.047153	0.038*	
H25C	0.427041	0.052451	0.066930	0.038*	

### 1-(4-Methylbenzenesulfonyl)-1-phenyl-1H,2H-cyclobuta[c]quinoline Atomic displacement parameters (Å^2^)

**Table:**

	*U*^11^	*U*^22^	*U*^33^	*U*^12^	*U*^13^	*U*^23^
S1	0.01716 (18)	0.01263 (18)	0.01518 (18)	−0.00107 (13)	0.00034 (13)	−0.00060 (13)
O1	0.0275 (6)	0.0139 (5)	0.0194 (5)	−0.0026 (4)	0.0024 (4)	0.0008 (4)
N1	0.0271 (7)	0.0199 (7)	0.0269 (7)	−0.0069 (6)	0.0049 (6)	−0.0025 (6)
O2	0.0176 (5)	0.0206 (6)	0.0213 (5)	−0.0007 (4)	−0.0014 (4)	−0.0010 (5)
C2	0.0201 (8)	0.0236 (8)	0.0249 (8)	−0.0050 (6)	0.0026 (6)	−0.0039 (7)
C3	0.0200 (7)	0.0185 (8)	0.0178 (7)	−0.0012 (6)	0.0023 (6)	−0.0030 (6)
C4	0.0201 (7)	0.0146 (7)	0.0149 (7)	−0.0022 (6)	0.0033 (5)	−0.0015 (5)
C5	0.0234 (8)	0.0146 (7)	0.0139 (7)	−0.0017 (6)	0.0031 (6)	−0.0019 (5)
C6	0.0247 (8)	0.0170 (7)	0.0156 (7)	−0.0005 (6)	0.0013 (6)	−0.0008 (6)
C7	0.0295 (9)	0.0222 (8)	0.0176 (7)	0.0048 (7)	0.0011 (6)	−0.0007 (6)
C8	0.0413 (10)	0.0173 (8)	0.0218 (8)	0.0044 (7)	0.0044 (7)	0.0028 (6)
C9	0.0387 (10)	0.0141 (8)	0.0251 (8)	−0.0047 (7)	0.0067 (7)	−0.0005 (6)
C10	0.0292 (8)	0.0149 (7)	0.0172 (7)	−0.0032 (6)	0.0053 (6)	−0.0029 (6)
C11	0.0167 (7)	0.0191 (8)	0.0212 (7)	0.0006 (6)	−0.0008 (6)	−0.0014 (6)
C12	0.0166 (7)	0.0136 (7)	0.0165 (7)	0.0007 (5)	0.0010 (5)	0.0003 (6)
C13	0.0169 (7)	0.0153 (7)	0.0173 (7)	−0.0017 (5)	0.0031 (5)	−0.0015 (6)
C14	0.0236 (8)	0.0172 (8)	0.0213 (7)	0.0020 (6)	0.0030 (6)	0.0007 (6)
C15	0.0281 (9)	0.0146 (8)	0.0305 (9)	0.0007 (6)	0.0074 (7)	−0.0010 (7)
C16	0.0281 (9)	0.0221 (9)	0.0251 (8)	−0.0061 (7)	0.0071 (7)	−0.0094 (7)
C17	0.0225 (8)	0.0245 (8)	0.0181 (7)	−0.0044 (6)	0.0032 (6)	−0.0025 (6)
C18	0.0197 (7)	0.0164 (7)	0.0189 (7)	−0.0012 (6)	0.0025 (6)	−0.0002 (6)
C19	0.0169 (7)	0.0144 (7)	0.0157 (7)	−0.0003 (5)	0.0008 (5)	−0.0021 (6)
C20	0.0223 (8)	0.0152 (7)	0.0195 (7)	0.0013 (6)	−0.0008 (6)	0.0010 (6)
C21	0.0235 (8)	0.0210 (8)	0.0176 (7)	0.0029 (6)	−0.0036 (6)	0.0001 (6)
C22	0.0165 (7)	0.0199 (8)	0.0201 (7)	−0.0009 (6)	0.0019 (6)	−0.0040 (6)
C23	0.0177 (7)	0.0144 (7)	0.0226 (7)	0.0009 (5)	0.0024 (6)	−0.0012 (6)
C24	0.0176 (7)	0.0167 (7)	0.0178 (7)	0.0021 (5)	0.0004 (5)	0.0009 (6)
C25	0.0262 (8)	0.0250 (9)	0.0247 (8)	0.0000 (7)	−0.0035 (7)	−0.0083 (7)

### 1-(4-Methylbenzenesulfonyl)-1-phenyl-1H,2H-cyclobuta[c]quinoline Geometric parameters (Å, º)

**Table:**

S1—O1	1.4402 (12)	C13—C14	1.393 (2)
S1—O2	1.4446 (11)	C13—C18	1.396 (2)
S1—C19	1.7579 (15)	C14—C15	1.389 (2)
S1—C12	1.8219 (15)	C14—H14	0.9500
N1—C2	1.323 (2)	C15—C16	1.388 (3)
N1—C10	1.374 (2)	C15—H15	0.9500
C2—C3	1.406 (2)	C16—C17	1.384 (2)
C2—H2	0.9500	C16—H16	0.9500
C3—C4	1.364 (2)	C17—C18	1.392 (2)
C3—C11	1.518 (2)	C17—H17	0.9500
C4—C5	1.406 (2)	C18—H18	0.9500
C4—C12	1.518 (2)	C19—C24	1.389 (2)
C5—C6	1.415 (2)	C19—C20	1.396 (2)
C5—C10	1.430 (2)	C20—C21	1.384 (2)
C6—C7	1.369 (2)	C20—H20	0.9500
C6—H6	0.9500	C21—C22	1.399 (2)
C7—C8	1.408 (3)	C21—H21	0.9500
C7—H7	0.9500	C22—C23	1.390 (2)
C8—C9	1.365 (3)	C22—C25	1.503 (2)
C8—H8	0.9500	C23—C24	1.386 (2)
C9—C10	1.418 (2)	C23—H23	0.9500
C9—H9	0.9500	C24—H24	0.9500
C11—C12	1.589 (2)	C25—H25A	0.9800
C11—H11A	0.9900	C25—H25B	0.9800
C11—H11B	0.9900	C25—H25C	0.9800
C12—C13	1.514 (2)		
			
O1—S1—O2	119.04 (7)	C4—C12—S1	112.56 (10)
O1—S1—C19	108.44 (7)	C11—C12—S1	114.56 (10)
O2—S1—C19	108.59 (7)	C14—C13—C18	119.23 (14)
O1—S1—C12	108.09 (7)	C14—C13—C12	119.61 (14)
O2—S1—C12	106.19 (7)	C18—C13—C12	121.15 (14)
C19—S1—C12	105.71 (7)	C15—C14—C13	120.48 (15)
C2—N1—C10	119.52 (14)	C15—C14—H14	119.8
N1—C2—C3	119.83 (15)	C13—C14—H14	119.8
N1—C2—H2	120.1	C16—C15—C14	119.97 (16)
C3—C2—H2	120.1	C16—C15—H15	120.0
C4—C3—C2	120.88 (15)	C14—C15—H15	120.0
C4—C3—C11	94.75 (13)	C17—C16—C15	119.95 (15)
C2—C3—C11	144.35 (15)	C17—C16—H16	120.0
C3—C4—C5	122.04 (15)	C15—C16—H16	120.0
C3—C4—C12	93.72 (13)	C16—C17—C18	120.30 (15)
C5—C4—C12	144.13 (14)	C16—C17—H17	119.8
C4—C5—C6	127.03 (14)	C18—C17—H17	119.8
C4—C5—C10	113.29 (14)	C17—C18—C13	120.04 (15)
C6—C5—C10	119.62 (15)	C17—C18—H18	120.0
C7—C6—C5	120.10 (15)	C13—C18—H18	120.0
C7—C6—H6	120.0	C24—C19—C20	121.45 (14)
C5—C6—H6	120.0	C24—C19—S1	119.72 (12)
C6—C7—C8	120.50 (16)	C20—C19—S1	118.82 (12)
C6—C7—H7	119.8	C21—C20—C19	118.39 (15)
C8—C7—H7	119.8	C21—C20—H20	120.8
C9—C8—C7	120.81 (16)	C19—C20—H20	120.8
C9—C8—H8	119.6	C20—C21—C22	121.36 (15)
C7—C8—H8	119.6	C20—C21—H21	119.3
C8—C9—C10	120.68 (16)	C22—C21—H21	119.3
C8—C9—H9	119.7	C23—C22—C21	118.75 (14)
C10—C9—H9	119.7	C23—C22—C25	120.80 (15)
N1—C10—C9	117.39 (15)	C21—C22—C25	120.45 (15)
N1—C10—C5	124.33 (15)	C24—C23—C22	121.10 (15)
C9—C10—C5	118.27 (16)	C24—C23—H23	119.4
C3—C11—C12	85.29 (11)	C22—C23—H23	119.4
C3—C11—H11A	114.4	C23—C24—C19	118.94 (14)
C12—C11—H11A	114.4	C23—C24—H24	120.5
C3—C11—H11B	114.4	C19—C24—H24	120.5
C12—C11—H11B	114.4	C22—C25—H25A	109.5
H11A—C11—H11B	111.6	C22—C25—H25B	109.5
C13—C12—C4	119.07 (13)	H25A—C25—H25B	109.5
C13—C12—C11	115.51 (12)	C22—C25—H25C	109.5
C4—C12—C11	86.19 (11)	H25A—C25—H25C	109.5
C13—C12—S1	107.93 (10)	H25B—C25—H25C	109.5
			
C10—N1—C2—C3	−1.4 (2)	C19—S1—C12—C13	−173.08 (10)
N1—C2—C3—C4	−1.5 (2)	O1—S1—C12—C4	169.44 (10)
N1—C2—C3—C11	176.2 (2)	O2—S1—C12—C4	−61.76 (12)
C2—C3—C4—C5	3.6 (2)	C19—S1—C12—C4	53.49 (12)
C11—C3—C4—C5	−175.11 (14)	O1—S1—C12—C11	73.08 (12)
C2—C3—C4—C12	−179.50 (15)	O2—S1—C12—C11	−158.12 (11)
C11—C3—C4—C12	1.81 (12)	C19—S1—C12—C11	−42.87 (12)
C3—C4—C5—C6	174.77 (15)	C4—C12—C13—C14	−153.41 (14)
C12—C4—C5—C6	0.0 (3)	C11—C12—C13—C14	−52.97 (19)
C3—C4—C5—C10	−2.5 (2)	S1—C12—C13—C14	76.70 (16)
C12—C4—C5—C10	−177.23 (19)	C4—C12—C13—C18	25.7 (2)
C4—C5—C6—C7	−176.19 (15)	C11—C12—C13—C18	126.14 (15)
C10—C5—C6—C7	0.9 (2)	S1—C12—C13—C18	−104.18 (15)
C5—C6—C7—C8	−1.7 (2)	C18—C13—C14—C15	0.8 (2)
C6—C7—C8—C9	0.9 (3)	C12—C13—C14—C15	179.89 (15)
C7—C8—C9—C10	0.6 (3)	C13—C14—C15—C16	−1.6 (3)
C2—N1—C10—C9	−176.19 (15)	C14—C15—C16—C17	1.0 (3)
C2—N1—C10—C5	2.4 (2)	C15—C16—C17—C18	0.3 (3)
C8—C9—C10—N1	177.31 (15)	C16—C17—C18—C13	−1.2 (2)
C8—C9—C10—C5	−1.4 (2)	C14—C13—C18—C17	0.6 (2)
C4—C5—C10—N1	−0.5 (2)	C12—C13—C18—C17	−178.52 (14)
C6—C5—C10—N1	−177.98 (14)	O1—S1—C19—C24	160.91 (12)
C4—C5—C10—C9	178.11 (14)	O2—S1—C19—C24	30.22 (15)
C6—C5—C10—C9	0.6 (2)	C12—S1—C19—C24	−83.38 (14)
C4—C3—C11—C12	−1.74 (12)	O1—S1—C19—C20	−18.36 (15)
C2—C3—C11—C12	−179.8 (2)	O2—S1—C19—C20	−149.06 (12)
C3—C4—C12—C13	115.45 (14)	C12—S1—C19—C20	97.34 (13)
C5—C4—C12—C13	−69.0 (3)	C24—C19—C20—C21	−0.1 (2)
C3—C4—C12—C11	−1.73 (12)	S1—C19—C20—C21	179.12 (12)
C5—C4—C12—C11	173.8 (2)	C19—C20—C21—C22	0.2 (2)
C3—C4—C12—S1	−116.78 (11)	C20—C21—C22—C23	0.0 (2)
C5—C4—C12—S1	58.8 (3)	C20—C21—C22—C25	179.90 (15)
C3—C11—C12—C13	−118.96 (13)	C21—C22—C23—C24	−0.2 (2)
C3—C11—C12—C4	1.56 (11)	C25—C22—C23—C24	179.92 (15)
C3—C11—C12—S1	114.66 (11)	C22—C23—C24—C19	0.2 (2)
O1—S1—C12—C13	−57.12 (12)	C20—C19—C24—C23	0.0 (2)
O2—S1—C12—C13	71.67 (11)	S1—C19—C24—C23	−179.29 (12)

### 1-(4-Methylbenzenesulfonyl)-1-phenyl-1H,2H-cyclobuta[c]quinoline Hydrogen-bond geometry (Å, º)

**Table:**

*D*—H···*A*	*D*—H	H···*A*	*D*···*A*	*D*—H···*A*
C23—H23···O2^i^	0.95	2.52	3.431 (2)	161
C24—H24···O1^i^	0.95	2.47	3.2357 (19)	137

Symmetry code: (i) −*x*+3/2, *y*−1/2, −*z*+1/2.
